# Initial experience, safety, and feasibility using remote access or onsite technical support for complex ablation procedures: results of the REMOTE study

**DOI:** 10.1093/ehjdh/ztae013

**Published:** 2024-02-19

**Authors:** Christian-H Heeger, Julia Vogler, Charlotte Eitel, Marcel Feher, Sorin Ștefan Popescu, Bettina Kirstein, Sascha Hatahet, Benham Subin, Karl-Heinz Kuck, Roland R Tilz

**Affiliations:** Department of Rhythmology, University Heart Center Lübeck, University Hospital Schleswig-Holstein, Ratzeburger Allee 160, D-23538 Lübeck, Germany; German Center for Cardiovascular Research (DZHK), Partner Site Hamburg/Kiel/Lübeck, Lübeck, Germany; Department of Rhythmology, Abteilung für Kardiologie & Internistische Intensivmedizin Asklepios Klinik Hamburg Altona, Paul-Ehrlich-Straße 1, 22763 Hamburg, Germany; Department of Rhythmology, University Heart Center Lübeck, University Hospital Schleswig-Holstein, Ratzeburger Allee 160, D-23538 Lübeck, Germany; Department of Rhythmology, University Heart Center Lübeck, University Hospital Schleswig-Holstein, Ratzeburger Allee 160, D-23538 Lübeck, Germany; Department of Rhythmology, University Heart Center Lübeck, University Hospital Schleswig-Holstein, Ratzeburger Allee 160, D-23538 Lübeck, Germany; Department of Rhythmology, University Heart Center Lübeck, University Hospital Schleswig-Holstein, Ratzeburger Allee 160, D-23538 Lübeck, Germany; Department of Rhythmology, University Heart Center Lübeck, University Hospital Schleswig-Holstein, Ratzeburger Allee 160, D-23538 Lübeck, Germany; Department of Rhythmology, University Heart Center Lübeck, University Hospital Schleswig-Holstein, Ratzeburger Allee 160, D-23538 Lübeck, Germany; Department of Rhythmology, University Heart Center Lübeck, University Hospital Schleswig-Holstein, Ratzeburger Allee 160, D-23538 Lübeck, Germany; Department of Rhythmology, University Heart Center Lübeck, University Hospital Schleswig-Holstein, Ratzeburger Allee 160, D-23538 Lübeck, Germany; Department of Rhythmology, University Heart Center Lübeck, University Hospital Schleswig-Holstein, Ratzeburger Allee 160, D-23538 Lübeck, Germany; German Center for Cardiovascular Research (DZHK), Partner Site Hamburg/Kiel/Lübeck, Lübeck, Germany

**Keywords:** Remote support, Electroanatomical mapping, Arrhythmias, Catheter ablation, Digital health, Telemedicine

## Abstract

**Aims:**

Electroanatomical mapping (EAM) systems are essential for the treatment of cardiac arrhythmias. The EAM system is usually operated by qualified staff or field technical engineers from the control room. Novel remote support technology allows for remote access of EAM via online services. Remote access increases the flexibility of the electrophysiological lab, reduces travel time, and overcomes hospital access limitations especially during the COVID-19 pandemic. Here, we report on the feasibility and safety of EAM remote access for cardiac ablation procedures.

**Methods and results:**

Mapping and ablation were achieved by combining the EnsiteX™ EAM system and the integrated Ensite™ Connect Remote Support software, together with an integrated audiovisual solution system for remote support (Medinbox). Communication between the operator and the remote support was achieved using an incorporated internet-based common communication platform (Zoom™), headphones, and high-resolution cameras. We investigated 50 remote access–assisted consecutive electrophysiological procedures from September 2022 to February 2023 (remote group). The data were compared with matched patients (*n* = 50) with onsite support from the control room (control group). The median procedure time was 100 min (76, 120; remote) vs. 86 min (60, 110; control), *P* = 0.090. The procedural success (both groups 100%, *P* = 0.999) and complication rate (remote: 2%, control: 0%, *P* = 0.553) were comparable between the groups. Travel burden could be reduced by 11 280 km.

**Conclusion:**

Remote access for EAM was feasible and safe in this single-centre study. Procedural data were comparable to procedures with onsite support. In the future, this new solution might have a great impact on facilitating electrophysiological procedures.

## Graphical Abstract

**Graphical Abstract:**
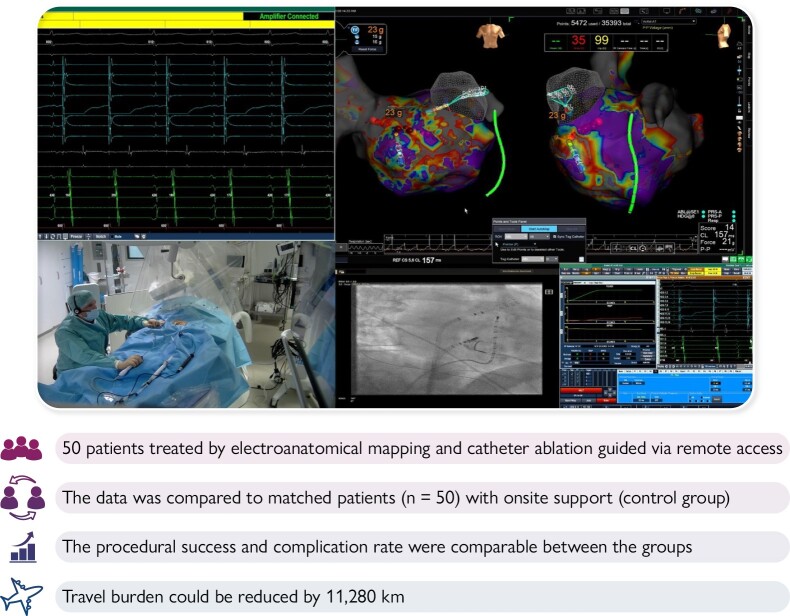

**What's new?**

- Electroanatomical mapping support, provided by a remote field engineer, seems to be feasible and safe.
- Additionally, real-time audiovisual communication via Medinbox during complex electrophysiological procedures through a stable internet connection is feasible and safe.
- Periprocedural parameters supported by remote staff are comparable to conventional onsite support.
- Remote access facilitates procedural flexibility, shares medical knowledge in real time, and provides educational opportunities not only for physicians but also for new and experienced trainees in mapping.
- Remote support reduces high travel burden.

## Introduction

Catheter ablation is a safe and effective strategy for the treatment of complex cardiac arrhythmias and is increasingly performed. In the modern electrophysiological laboratory (EP lab), catheter ablation is usually guided by a 3D electroanatomic mapping (EAM) system navigated and supported by an onsite field technical engineer (FTE) with high-skilled and trained technical and medical knowledge.^1–3^ A good teamwork and communication between the operator and the FTE are essential for safe, effective, and efficient catheter ablation procedures. During the COVID-19 pandemic, travel restrictions limited availabilities of onsite FTE for EP labs.^4,5^ Additionally, a general shortage of skilled personnel in health care systems results in reduced procedure numbers. The consequences of this shortage impact routine elective procedures as well as emergency catheter ablation procedures (e.g. ablation of incessant ventricular tachycardia).^6^ Remote support and remote access of complex arrhythmia ablation procedures via a broadband internet–based audiovisual communication software in combination with an EAM system might overcome those issues. Until today, only very limited data in a low number of patients are available on remote support for cardiac ablation procedures.^7^

In the REMOTE study, we aim to assess the safety and feasibility of remote support via remote access in comparison to direct onsite technical support of EAM in complex cardiac arrhythmia ablation procedures.

## Methods

### Inclusion and exclusion criteria

This is a prospective, non-randomized, single-centre study (REMOTE study). Since October 2021, consecutive patients with the EnsiteX™ EAM and Ensite™ Connect Remote Support software-based remote access were included. Prospectively enrolled previous patients treated by onsite EnsiteX™ EAM support were used as a matched control group. The matching was based on the type of procedure and derived from a local prospective database (Lübeck ablation registry). Ablation procedures were 20 pulmonary vein isolation (PVI) (3 paroxysmal atrial fibrillation [PAF], 17 persistent atrial fibrillation [PersAF]), 4 CTI, 13 AT, 1 premature ventricular contracions (PVC), 3 ventricular tachycardia (VT), 2 atrio ventricular reentry tachycardia (AVRT), and 7 atrio ventricular nodule reentry tachycardia (AVNRT)/supra ventricular tachycardia (SVT) cases. The data were compared with 50 matched patients with previously performed electrophysiological procedures with EAM (EnsiteX™) and ablation guided by onsite FTE. The matching was based on the following criteria: onsite EnsiteX™ supported procedure before October 2021 and similar ablation procedures based on the ablation targets as the remote group. Exclusion criteria were patients not willing to participate in the Lübeck ablation registry and patients not eligible for catheter ablation procedures. All patients provided written informed consent before the procedure. All patient-related data were anonymized. The REMOTE study was part of the Lübeck ablation registry and approved by the local ethics committee (Lübeck ablation registry ethical review board number: WF-028/15) and was performed in accordance with the ethical standards laid down in the 1964 Declaration of Helsinki and its later amendments.

### Preprocedural management

Preprocedural transoesophageal echocardiography (TEE) was performed to rule out intracardiac thrombi before the procedure. No additional preprocedural imaging was carried out. In patients under the treatment with vitamin K antagonists (VKAs), the procedure was conducted under therapeutic international normalized ratio (INR) values between 2 and 3, while in those under non-VKA oral anticoagulants (NOACs), the morning dose of NOAC was omitted on the day of the procedure.

### Intraprocedural management

All procedures were performed by four physicians, who were highly experienced in electrophysiological procedures. A comprehensive description of the intraprocedural management has been reported in previous publications from our department.^2,8^ Briefly, all procedures were performed under deep sedation using midazolam, fentanyl, and continuous infusion of propofol following the recommendations of a position paper by the German Cardiac Society.^9^ For venous access, ultrasound-guided right femoral vein punctures were carried out, and 8 French (F) short sheaths were inserted. One 7 F diagnostic catheter was positioned within the coronary sinus (CS) via a sheath in the right femoral vein. For patients with left atrial (LA) access, single or double transseptal puncture (TSP) was performed under fluoroscopic guidance using a modified Brockenbrough technique and an 8.5 F transseptal sheath (SL1; St Jude Medical, Inc.). The LA sheaths were continuously flushed with heparinized saline (20 mL/h). In cases with LA or left ventricular access, an activated clotting time (ACT) of >300 s was targeted by means of heparin boluses.

### Remote support and onsite support

An internet-based communication platform (Zoom Video Communications, Inc.) in combination with a broadband internet connection was used to establish an audiovisual connection between the EP lab and the FTE based at home office in Germany. Medinbox (Abbott) was used as interface for the audiovisual communication. The Medinbox was equipped with a high-resolution camera, loudspeakers, Bluetooth headphones, and microphones. After a stable connection was established, EAM was performed with EnsiteX™ (Abbott) by utilizing remote support via Ensite™ Connect Remote Support only. Via Medinbox, audiovisual signals (fluoroscopic, EnsiteX™, ECG, and recordings) were transferred in real time. The FTE had remote access to the EnsiteX™ EAM system, and no remote support of the recording system was possible. For onsite support, the procedures were guided by an onsite FTE. In this case, the communication with the operator was achieved using radio-based headphones. All procedures were conducted using the EnsiteX™ EAM system.

**Figure 1:**
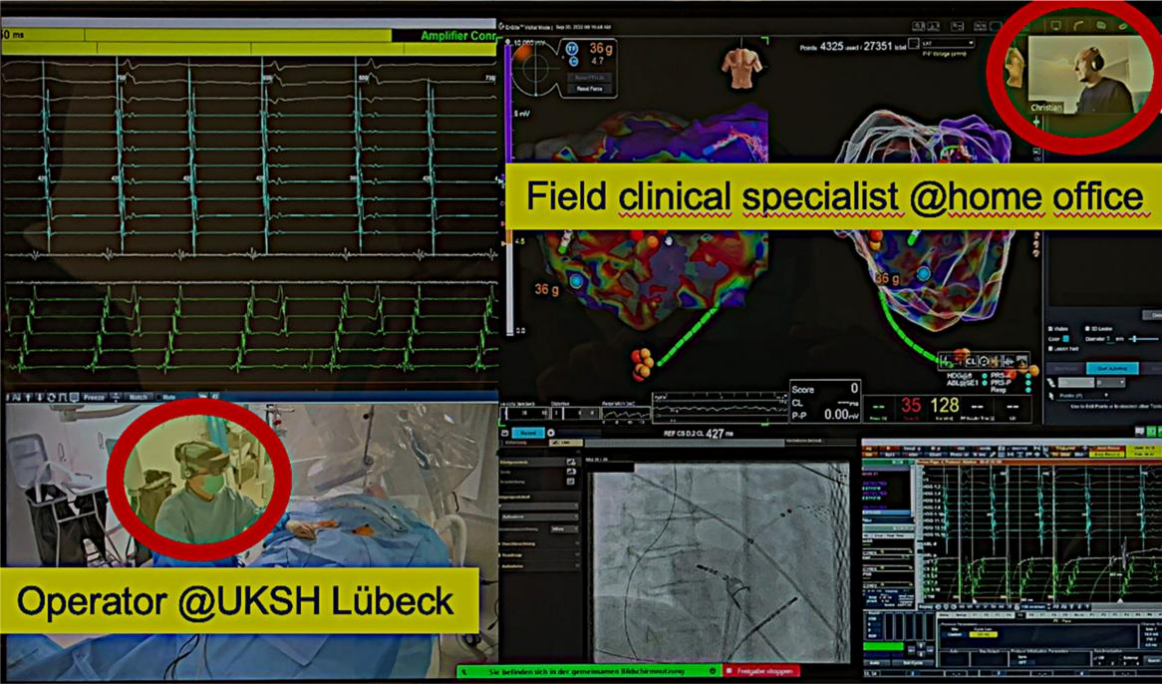
Screen of the Medinbox at the electrophysiological lab with integrated visualization of electrograms, electroanatomical mapping, fluoroscopy, and high-definition camera images of the electrophysiological lab and the remote support via a field clinical specialist from home office with 290 km distance. Remote access–guided mapping and ablation of an atrial tachycardia originating within the left atrium.

**Figure 2:**
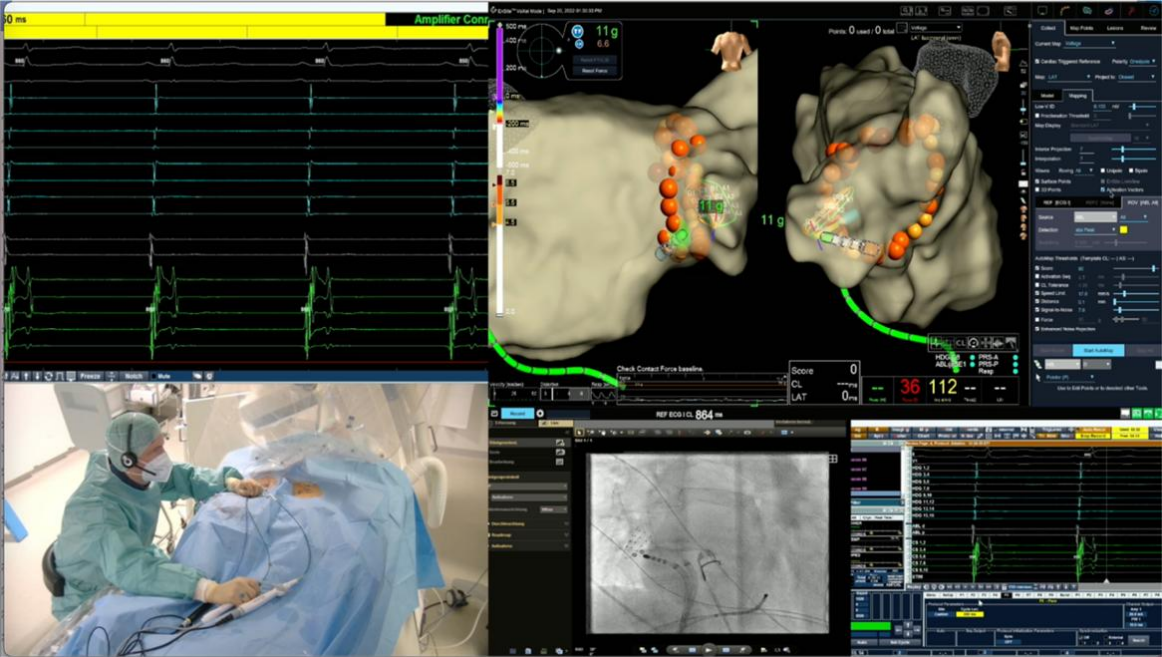
View of the field technical engineer at home office. Please see the integrated visualization of electrograms, electroanatomical mapping system, fluoroscopy, and high-definition camera images. Remote access–guided mapping and ablation of atrial fibrillation by pulmonary vein isolation.

In case of a lost internet connection, the following back-up plan was conducted. There were always physicians in the lab who were able to support the 3D mapping system. Additionally, all remote support FTEs were available via telephone calls for troubleshooting. No additional training plan was conducted. *Figure 1* shows the screen of the Medinbox at the EP lab, and *Figure 2* shows the screen of the FTE at home office.

### Postprocedural care

A ‘figure-of-eight’ suture and a pressure bandage were used to prevent femoral bleeding.^10^ The bandage was removed 4 h after the procedure and the suture on the next day. Pericardial effusion (PE) was ruled out in all patients, using transthoracic echocardiography (TTE) performed immediately, at 2 h, and on the first postprocedural day. For patients under VKA treatment and with subtherapeutic INR values, low-molecular-weight heparin (LMWH) was administered until a therapeutic INR of 2–3 was achieved. Non-VKA oral anticoagulants were reinitiated the evening after the procedure. All patients received oral anticoagulation for at least 3 months postprocedural, which was thereafter continued based on the individual thromboembolic risk (CHA_2_DS_2_-VASc score). All patients received an antiarrhythmic drug for 3 months post ablation, while a proton pump inhibitor (PPI) was prescribed for 6 weeks. Following a 3-month blanking period, patients completed outpatient clinic visits, including ECG and 24-h Holter ECG at 3, 6, and 12 months. In addition, regular telephone interviews were performed. Additional outpatient clinic visits were immediately initiated in cases of symptoms suggestive of arrhythmia recurrence.

### Endpoints

#### Primary endpoint

The primary efficacy endpoint was defined as reaching the target and goal of the catheter ablation procedure. This was: atrial fibrillation (AF): PVI (evidence of entrance block verified by a multipolar mapping catheter), typical atrial flutter: CTI (evidence of bidirectional block), AT (non-inducibility of the clinical AT), PVC: loss of PVC after catheter ablation, VT: non-inducibility of the clinical VT, AVRT: loss of preexcitation, and AVNRT: at least evidence of slow pathway modulation.

#### Secondary endpoints

The secondary endpoints were as follows: procedural parameters (e.g. procedure time, LA dwelling time, and fluoroscopy time), number and duration of radiofrequency (RF) applications, and number of first pass isolations as well as periprocedural complications. Periprocedural complications were defined according to the latest guidelines. Adverse events were divided into the following categories: possible, probable, or definitely related to the ablation procedure and were mentioned as safety events. An adverse event was considered serious if it resulted in a permanent injury, disability, or death; requiring an interventional treatment; or additional hospitalization for >24 h. All other safety events were defined as minor complications.

### Statistical analysis

Categorical variables are reported as absolute and relative frequencies, *n* (%). They were compared using the *χ*^2^ test or Fisher’s exact test as appropriate. The continuous variables were tested for normality using the Shapiro–Wilk test. They were reported as mean and standard deviation (SD) in case of normal distribution or as median and interquartile range (first quartile, third quartile) otherwise. They were compared using Welch’s *t*-test or the Mann–Whitney *U* test as appropriate. All *P*-values are two-sided, and a *P* < 0.05 was considered statistically significant. All statistical analyses were performed using the SPSS version 29.0 (IBM SPSS Statistics).

## Results

### Patient characteristics

A total of 50 consecutive patients underwent remote access–guided electrophysiological procedures with EAM (EnsiteX) and ablation. Patient baseline characteristics are shown in *Table 1*. No demographic differences were detected between the groups. The same four FTE performed the onsite and remote support.

**Table 1:** Baseline characteristics

Variable	Remote	Onsite	*P*
Patients	50	50	
Age, years	66.5 (56, 76)	66.5 (54, 78)	0.764
Female, %	44	40	0.685
BMI, kg/m^2^	25.4 (22, 30)	28.2 (24, 30)	0.857
Chronic heart failure, *n* (%)	6 (12)	4 (8)	0.505
Arterial hypertension, *n* (%)	35 (70)	34 (68)	0.829
Diabetes mellitus type II, *n* (%)	12 (12)	11 (11)	0.812
Coronary artery disease, *n* (%)	8 (16)	12 (24)	0.317

Values are counts, *n* (%), or median (first quartile, third quartile).

BMI, body mass index.

### Procedural characteristics

Procedural data are summarized in *Table 2*. Mean procedure time, ablation time, and procedural success were similar in both groups. All procedures were guided by EnsiteX EAM and related multielectrode mapping catheters (Advisor™ HD Grid Mapping Catheter, Sensor Enabled™ and Advisor™ Mapping Catheter, and Sensor Enabled™).^11^ For the remote group, all settings as well as the complete 3D mapping and analysis have been performed via remote access. For PVI, an EAM of the LA was performed after double TSP utilizing a multielectrode mapping catheter. Afterwards, the pulmonary vein ostium was tagged and ablated via a circumferential ablation line around the ipsilateral pulmonary veins. For patients with typical atrial flutter, an EAM map of the right atrium was performed via the ablation catheter, and the cavotricuspid isthmus was ablated utilizing RF energy. For patients with AT, a double TSP was performed, and an EAM of the LA was conducted utilizing a multielectrode mapping catheter. Afterwards, the local activation time map was analysed and interpreted to conduct an ablation strategy. For PVC mapping, the Advisor™ HD Grid Mapping Catheter was utilized to create an EAM of the right or left ventricle, and the earliest activation of the PVC was detected and ablated utilizing RF energy. For VT patients, we aimed to create a local activation time mapping of the clinical VT after induction via programmed ventricular stimulation. Afterwards, the local activation time map was analysed and interpreted to conduct an ablation strategy and to ablate all late potentials. The endpoint was non-inducibility of the clinical VT. Patients with AVNRT received a 3D EAM of the right atrium, right ventricle, and CS. The His bundle was tagged, and the slow pathway was ablated by RF energy. The patients with accessory pathways received a 3D EAM of the tricuspid or mitral annulus by annotation mapping, and the earliest ventricular activation site was ablated by RF energy. For catheter ablation, TactiCath™, TactiFlex™, and Flexibility™ ablation catheters were used.^12–14^ In patients treated by PVI, a high-power short-duration ablation protocol was used.^2,3,14,15^

**Table 2:** Procedural details

Variable	Remote	Onsite	*P*
Number of patients	50	50	
Total procedure time, min	100 (76, 120)	86 (60, 110)	0.090
Total fluoroscopy time, min	9.1 (6, 13)	8.7 (5, 12)	0.225
Total amount of contrast agent, mL	40 (1, 50)	40 (0, 50)	0.853
Radiofrequency applications, *n*	55 (33, 106)	44 (26, 79)	0.371
Indication for catheter ablation			
Typical atrial flutter, *n* (%)	4 (8)	4 (8)	0.999
Atrial fibrillation, *n* (%)	20 (40)	20 (40)	0.999
Atrial tachycardia, *n* (%)	13 (26)	13 (26)	0.999
Premature ventricular contraction, *n* (%)	1 (2)	1 (2)	0.999
Ventricular tachycardia, *n* (%)	3 (6)	3 (6)	0.999
Supraventricular tachycardia (AVNRT), *n* (%)	7 (14)	7 (14)	0.999
Supraventricular tachycardia (AVRT), *n* (%)	2 (4)	2 (4)	0.999
Periprocedural complications			
Major complications, *n* (%)	1 (2)	0 (0)	0.553
Cardiac tamponade, *n* (%)	0	0	0.999
Severe bleeding, *n* (%)	0	0	0.999
Phrenic nerve injury, *n* (%)	0	0	0.999
Stroke or TIA, *n* (%)	0	0	0.999
AV-III° block, *n* (%)	1 (2)	0	0.553
Minor complications, *n* (%)	0 (0)	2 (4)	0.771
Minor bleeding, *n* (%)	0 (0)	2 (4)	0.771
Pericardial effusion, *n* (%)	0	0	0.999
Transient air embolism, *n* (%)	0	0	0.999
SA arrest, *n* (%)	0	1 (2)	0.533
Aspiration, *n* (%)	0	1 (2)	0.533

Values are counts, *n* (%), or median (first quartile, third quartile).

Acute procedural success was obtained in all cases. Median procedure time in the remote and onsite groups were 100 min (76, 120) and 86 min (60, 110), respectively (*P* = 0.090). Median fluoroscopy time in the remote and control groups were 9.1 min (6, 13) and 8.7 min (5, 12), respectively (*P* = 0.225). One patient experienced transient complete atrioventricular (AV) block, and one had a sinoatrial (SA) block. The complications were deemed to be unrelated to the remote support.

### Remote access experience

In all remote cases (50/50), remote access success rate was 100% with no technical issues. There were no technical concerns with the Medinbox system reported. The EAM was stable, and there was no delay in mapping guidance or mapping editing (no map shift, software crash). Connectivity was stable throughout all the procedures. In a few cases, the Bluetooth headphones had to be switched due to empty batteries after several hours of usage. There was no need for switch to onsite FTE support in any case. The remote access as well as onsite support was performed by four different FTEs. In 11 remote access days, 2 procedures were guided by remote access, and in 1 remote access day, 3 procedures were also guided by remote access.

### Travel burden saved by remote access

Due to remote access by the FTE, time-consuming travel to the procedure performing clinic became dispensable. The geographical distances between the remote support FTE and the clinic are 70 km (FTE: 1), 163 km (FTE: 2), 206 km (FTE: 3), and 290 km (FTE: 4). The individual number of remote access–guided procedures, guided procedures per travel day, total remote access days, and total saved travel burden are mentioned in *Table 3*. All four FTEs were travelling by car. Calculations show a reduction of cumulative travel distance for these 50 remote cases of 11 280 km. Based on a diesel consumption of 5 L/100 km, a calculation of the hypothetical CO_2_ emission was conducted. A total of 2.6 (0.03, 0.1) tons of CO_2_ could be saved by remote access. It is important to mention that the production, installation, and electricity usage of remote-based equipment is also producing CO_2_ emissions, which were not calculated.

**Table 3:** Travel burden saved by remote access

Variable	Guided procedures, *n*	Guided procedures/travel day, *n*	Total saved travel days, *n*	Individual travel distance per procedure, km	Total saved travel burden, km	Total saved CO_2_, t
FTE 1	19	1.1	17	140	2380	0.56
FTE 2	9	1.5	6	326	1956	0.38
FTE 3	11	1.6	7	412	2884	0.67
FTE 4	11	1.6	7	580	4060	0.95
All	50	1.4	37	—	11 280	2.6

FTE, field technical engineer; km, kilometre; t, tons; CO_2_, carbon dioxide.

## Discussion

Here, we are presenting the first prospective data on remote support of EAM during electrophysiology procedures via remote access by a FTE.

The main findings are the following:

1. Electroanatomical mapping by a remote FTE via remote access was feasible and safe.
2. Additionally, real-time audiovisual communication via Medinbox during complex EP procedures through a stable internet connection was feasible and safe.
3. Periprocedural parameters supported by remote staff are comparable to conventional onsite support.
4. Remote support reduces high travel burden.

Until today, only very limited data in a low number of patients are available on remote support for cardiac ablation procedures.^7^ Travel restrictions during the COVID-19 pandemic limited availabilities of onsite FTE for EP labs.^4^ Additionally, there is a world-wide shortage of skilled personnel in the health care systems, which is resulting in limited capacity of medical interventions and surgery procedures in general.^6^ Furthermore, adverse weather conditions might limit the travel abilities in many countries. Remote support of EAM via broadband internet–based audiovisual communication software might be an option for above-mentioned issues.

Here, the success rate of the procedures with remote access was 100%. Remote access of EAM in the treatment of all atrial and ventricular procedures was performed without any difficulties and could easily be implemented in our EP programme.

Although there was no statistical difference between the two groups concerning mean procedure time, the *P*-value was borderline (*P* = 0.090). This observation might be driven by a prolonged setting up time for the Medinbox and Zoom settings in the remote arm. However, the 50 reported cases are the first cases utilizing remote access in our set-up; therefore, a particular learning curve might be the reason for this observation.

The comparison to the onsite-guided EAM procedures found no differences concerning the patients’ characteristics and periprocedural data. However, there was a trend towards a longer procedure time in the remote group. There was no significant difference in the rate of complication between the remote and onsite groups. This observation is in line with previous studies on remote support, and no complication was related to the remote support.^7,16^

Since it was never necessary to switch to an onsite support, it seems not to be necessary to provide an onsite support during remote support cases for safety reasons as it was recently described by Müssigbrodt *et al.*^7^ A stable internet connection is necessary to provide safe and feasible remote support via remote access. The usage of an audiovisual communication platform via internet might be raising data safety aspects. However, complete security against cyberattacks cannot be provided by any software. The Medinbox system is limiting those issues by deleting all patient-related data automatically. Furthermore, the Zoom software features encrypted data and may be considered as safe.

We were able to demonstrate that remote access of EP procedures was able to reduce travel burden for FTE. The calculations show a reduction of cumulative travel distance by a total of 11 280 km. With remote access, 2.6 tons of CO_2_ could be saved.

Based on a stable internet connection, remote support seems to be possible from every position world-wide not only of the FTE but also for the EP lab. This aspect was recently shown by Müssigbrodt *et al.*^7^ with an EP lab localized at the University Hospital of Martinique (French overseas territory, Caribbean region) and remote support by an FTE based in mainland France, about 7000 km across the Atlantic Ocean. Although the travel distances were less in our study, this aspect should be an important factor for cost and carbon dioxide emission reduction. Additionally, the home office environment for the FTE might have a positive social effect on the one hand, yet on the other hand, forcing the FTE to work remotely might result in social isolation. More effective operational availability is only one aspect for the providing vendor.

Remote access is a promising novel technology for guiding electrophysiological procedures. Additionally, remote access might provide educational opportunities, individual case interpretation by experts in the field of electrophysiology, and reduction of the lab downtime. In the future, this new solution might have a great impact on facilitating electrophysiological and other interventional procedures.

### Limitations

This study is the first prospective analysis on remote access–guided support of EAM procedures for the treatment of cardiac arrhythmias. It is a non-randomized analysis resulting in potential biases. Although we are presenting single-centre experience with a relatively small number of patients, consecutive patients were prospectively evaluated, and all procedures were performed by four highly experienced operators. However, the impact on safety and efficacy cannot be completely assessed in this relatively small population. We are not able to exclude rare adverse events. Therefore, the findings should be interpreted with caution and only hypothesis generating.

## Conclusions

Remote access for EAM of cardiac arrhythmias seems to be feasible and safe in this single-centre study. Procedural data were comparable to onsite-supported procedures. Travel burden could be reduced by >11 000 km in 50 electrophysiological cases.

## Data Availability

Non-digital data supporting this study are curated at the study centre of the Department of Rhythmology, University Hospital Schleswig-Holstein, Germany.
