# Correction to: Initial experience, safety, and feasibility using remote access or onsite technical support for complex ablation procedures: results of the REMOTE study

**DOI:** 10.1093/ehjdh/ztae056

**Published:** 2024-07-29

This is a correction to: Christian-H Heeger, Julia Vogler, Charlotte Eitel, Marcel Feher, Sorin Ștefan Popescu, Bettina Kirstein, Sascha Hatahet, Benham Subin, Karl-Heinz Kuck, Roland R Tilz, Initial experience, safety, and feasibility using remote access or onsite technical support for complex ablation procedures: results of the REMOTE study, *European Heart Journal - Digital Health*, Volume 5, Issue 3, May 2024, Pages 356–362, https://doi.org/10.1093/ehjdh/ztae013

The **Funding** section of the originally published manuscript has been corrected to read as follows:

“Funding von Abbott Medical zur Open Access Publication.”

